# Base excision repair and its implications to cancer therapy

**DOI:** 10.1042/EBC20200013

**Published:** 2020-07-10

**Authors:** Gabrielle J. Grundy, Jason L. Parsons

**Affiliations:** Cancer Research Centre, Department of Molecular and Clinical Cancer Medicine, University of Liverpool, 200 London Road, Liverpool, L3 9TA, U.K.

**Keywords:** Base excision repair, Cancer therapy, DNA damage, DNA repair

## Abstract

Base excision repair (BER) has evolved to preserve the integrity of DNA following cellular oxidative stress and in response to exogenous insults. The pathway is a coordinated, sequential process involving 30 proteins or more in which single strand breaks are generated as intermediates during the repair process. While deficiencies in BER activity can lead to high mutation rates and tumorigenesis, cancer cells often rely on increased BER activity to tolerate oxidative stress. Targeting BER has been an attractive strategy to overwhelm cancer cells with DNA damage, improve the efficacy of radiotherapy and/or chemotherapy, or form part of a lethal combination with a cancer specific mutation/loss of function. We provide an update on the progress of inhibitors to enzymes involved in BER, and some of the challenges faced with targeting the BER pathway.

## Introduction

### Overview of the BER pathway

**Figure 1:**
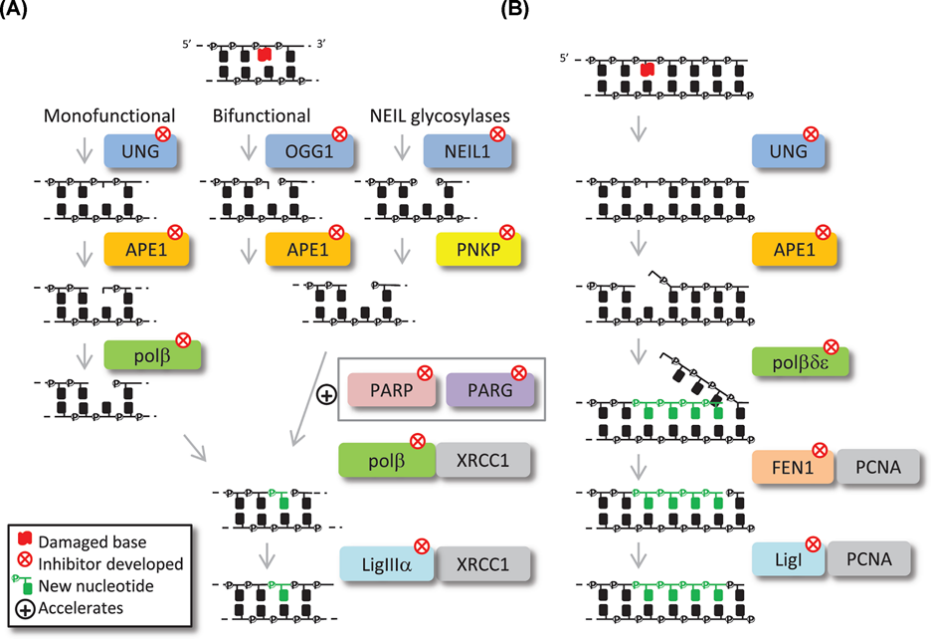
Overview of SP-BER and LP-BER (**A**) Base damage (red flag) is recognised by one of 11 damage-specific DNA glycosylases that are monofunctional (removes base creating an AP site); bifunctional (removes base and cleaves phosphodiester backbone 3′ to the lesion) or Nei-like (cleaves phosphodiester bond either side of lesion). APE1 incises the AP site, or removes the 3′-α,β-unsaturated aldehyde remaining from bifunctional DNA glycosylase action. PNKP is required to remove 3′-phosphate termini following Nei-like DNA base excision. At this stage, PARP1 recognises SSB/gap intermediates protecting these from degradation, and facilitates repair through protein recruitment. Polβ through its lyase activity excises the 5′-dRP moiety, and simultaneously fills the one nucleotide gap (green nucleotide). XRCC1-LigIIIα complex interacts with Polβ and repairs the remaining nick in the DNA, thus completing SP-BER. (**B**) When the 5′-DNA end is not amenable to Polβ, LP-BER is employed. A polymerase switch to Polδ/ε stimulates strand displacement and creates a 2–8 nucleotide 5′-flap. FEN1 cleaves the flap and LigI ligates the subsequent nick, both of which are stimulated by the PCNA clamp slider. Each enzymatic step of the pathway can be targeted by small molecule inhibitors, as indicated by the red cross.

Base excision repair is a highly conserved mechanism dealing with oxidative damage generated by respiration, natural hydrolysis and alkylation reactions that occur in each cell, many thousands of times a day [1]. In humans at least 30 proteins are involved in both short patch repair (SP-BER), the removal of a single non-bulky damaged base; and long patch repair (LP-BER), where 2–8 nucleotides are synthesised to displace the damaged area (Figure 1). The first step of the BER pathway is the recognition and removal of base damage by damage-specific DNA glycosylases [2]. Humans have 11 DNA glycosylases that can be subdivided into three groups (Table 1): (1) Monofunctional enzymes which excise the damaged base leaving an apurinic/apyrimidinic (AP) site and an intact phosphodiester backbone; (2) Bifunctional glycosylases that remove the base and cleave the phosphodiester bond on the 3′ side of the damaged base creating an 3′-α,β-unsaturated aldehyde (β-elimination); (3) Nei-like DNA glycolysases (NEIL) that can catalyse a β/δ-elimination reaction where the phosphodiester bond is cleaved either side of the removed lesion.

**Table 1:** DNA glycosylases: substrates, inhibitors and synthetic lethal partners

DNA glycosylase	Substrate	Inhibitor	Synthetic lethal partner
*Monofunctional*			
**UNG** Uracil DNA glycosylase	Uracil		APOBEC3B
**SMUG1** Single-strand selective monofunctional uracil DNA glycosylase	Uracil, 5-formyluracil, 5-hydroxyuracil and 5-hydroxymethyluracil		
**TDG** G/T Mismatch specific thymine DNA glycosylase	5-Formylcytosine and 5-carboxylcytosine in CpG. G:T and U:T mismatches		
**MBD4** Methyl-CpG binding domain protein 4	G:T mismatches within methylated and unmethylated CpG sites. Uracil or 5-fluorouracil in G:U mismatches		
**MPG** N-methylpurine DNA glycosylase	3-Methyladenine and 7-methylguanine		
**MUTYH** Adenine DNA glycosylase	7,8-Dihydro-8-oxoguanine (8-oxoG):adenine		
*Bifunctional*			
**NTH1** Endonuclease III-like protein 1	Oxidised pyrimidines, thymine glycol 8-OxoG		
**OGG1**8-oxoguanine DNA glycosylase 1	8-OxoG and formamidopyrimidine(Fapy)G	O8SU0268TH5487	MMR deficiency
*Endonuclease VIII-like*			
**NEIL1**	Thymine glycol, Fapy and 5-hydroxyuracil	2TX	FANCG
**NEIL2**	5-Hydroxyuracil		
**NEIL3**	Spiroiminodihydantoin and guanidinohydantoin		

Following monofunctional DNA glycosylase action, AP endonuclease 1 (APE1) recognises the AP site and hydrolyses the DNA backbone forming a single strand break (SSB) with a 5′-deoxyribosephosphate (dRP) and 3′-hydroxyl ends [3,4]. It also acts on the products of bifunctional glycosylases, where hydrolysis expels the 3′-α,β-unsaturated aldehyde and creates a one nucleotide gap product. The product of the NEIL glycosylases contains a 3′-phosphate group which requires polynucleotide kinase phosphatase (PNKP) to generate a 3′-hydroxyl end that is suitable for DNA polymerase action [5]. At this point, on the formation of a gap or SSB, poly(ADP-ribose) polymerase 1 (PARP1) is engaged which protects the strand break [6] and also plays a role in protein recruitment through its associated poly(ADP-ribosyl)ation activity. The major polymerase employed during BER is DNA polymerase β (Polβ), which fills the gap but also catalyses a lyase reaction that removes the 5′-dRP that may be present [7,8]. Finally, DNA ligase IIIα (LigIIIα) in complex with X-ray repair cross-complementing protein 1 (XRCC1) completes the process of SP-BER (Figure 1A) [9,10]. LP-BER is employed when the 5′-DNA ends are not amenable to Polβ action. Here, a polymerase switch occurs and a flap of 2–8 nucleotides are synthesised by DNA polymerases δ/ε (Polδ/ε), which associates with proliferating cell nuclear antigen (PCNA), displacing the damaged strand. Flap endonuclease 1 (FEN1) activity removes the displaced strand leaving a ligatable nick for DNA ligase I (LigI), which associates with the PCNA clamp slider (Figure 1B) [11,12].

### BER dependence following radiotherapy and chemotherapy

Anti-neoplastic drugs inhibit mitosis and many of them do so through alterations to DNA, which normally would be repaired by cells, but overwhelms rapidly dividing cells to trigger cell death. Such classes of chemotherapy agent include: nucleoside analogues that become incorporated into DNA (e.g. 5-fluorouracil; 5-FU) [13]; antifolates that inhibit the synthesis of deoxythymidine triphosphate and so increase uracil incorporation (e.g. pemetrexed) [14]; demethylating agents that cause DNA damage by trapping DNA methyltransferases (e.g. decitabine 5-aza-2′-deoxycytidine; 5-azadC) [15]; platinum drugs (e.g. cisplatin) [16,17]; and alkylating agents that produce DNA adducts (e.g. temozolomide; TMZ). Thus, several chemotherapy agents produce DNA modifications that rely on BER for removal and cytotoxicity in cancer cells. Current external (photon and particle beam) and internal radiotherapy approaches also generate a large proportion of DNA damage that is a target for BER. Ionising radiation emanating from these sources either directly or indirectly creates a mixture of base damage, oxidative damage and SSBs that are recognised by the BER pathway. The amount of DNA damage caused by chemotherapy and/or radiotherapy should overwhelm the cancer cell’s capacity for repair for therapeutic effectiveness.

## Inhibitors to the BER enzymes

One strategy for cancer therapy is to target BER enzymes with inhibitors, and in combination with radiotherapy and/or chemotherapy, this will create additional damage that exceeds the BER ability of the cancer cells. This is particularly important for specific tumour types that contain BER gene and protein overexpression. Another desirable strategy is where targeting BER enzymes can lead to specific killing of cancer cells via a synthetic lethal partnership, and where a tumorigenic mutation becomes reliant on BER to survive. In this section, we review the progress made with developing and characterising inhibitors to several BER enzymes using some of these strategies (and summarised in Tables 1 and 2).

**Table 2:** Targeting BER intermediates and enzymes

Target	Inhibitor	Synthetic lethal partner
Apurinic/apyrimidinic site	MX (TRC102)	
*BER enzymes*		
**APE1**		
lyase activity	CRT0044876	PTEN
	API3	BRCA1
		BRCA2
		ATM
Redox function	E3330	
	Gossypol/AT101	
**PNKP**	A12B4C3	PTEN
		SHP1
**FEN1**	NSC-281680	BRCA1
	SC13	BRCA2
	FENi#2	CDC4
		MRE11
**Polβ**	NSC666715	
	Pro13	
	Natamycin	
**LigIIIα/LigI**	L67	
**LigI**	L82	
**PARP1/PARP2**	Olaparib	BRCA1
	Veliparib	BRCA2
	Talazoparib	FANCG
	Niraparib	
	Rucaparib	
**PARG**	PDD00017272	BRCA1
	PDD00017273	BRCA2
	COH34	PALB2
	JA2-4/JA2131	FAM175A
		BARD1

### UNG inhibitors

In humans, there are two isoforms of uracil DNA glycosylase (UNG) that differ in localisation, as UNG1 is mitochondrial whereas UNG2 is nuclear, and these enzymes recognise U:A and U:G pairs in double-stranded DNA. When there are high levels of uracil incorporation in DNA, UNG activity is toxic as repeated attempts to excise the lesion result in an increase in strand breaks. The folate analogue pemetrexed, which inhibits thymidylate synthase to decrease dTTP levels and thus increases uracil misincorporation, is particularly effective in UNG-deficient colon and lung cancer cell lines [14]. Indeed, drug resistance can be induced by up-regulation of UNG expression, and pemetrexed sensitivity restored by using methoxyamine (MX), another BER inhibitor [14,18]. Thus, inhibiting BER could prevent the development of tumours resistant to folate analogues.

Targeting UNG has been suggested to cause synthetic lethality in the many cancer cells that have high APOBEC3B levels (e.g. bladder, cervix, lung, breast, and head and neck cancers) [19]. APOBEC3B is a cytosine deaminase that converts cytosine to uracil and causes an accumulation of C to T signature mutations in cancer genomes. A knockout of UNG has been demonstrated to kill APOBEC3B expressing cells due to an accumulation of uracil lesions in a mechanism dependent on non-canonical mismatch repair (MMR) [20]. Consequently, there is a need to develop specific small molecule inhibitors to UNG. Progress has been made from alkylated uracils that attach to the enzyme active site with sub-micromolar IC_50_ [21], to identifying small molecule inhibitors using uracil substrate fragment-linked to a library of aldehyde tethers [21,22]. Disappointingly, successful potency in cancer cell lines has not been reported with the UNG inhibitors and their action has only been demonstrated in cell free systems.

### OGG1 inhibitors

The bifunctional N-glycosylase/DNA lyase, 8-oxoguanine DNA glycosylase 1 (OGG1) recognises and removes 7,8-dihydro-8-oxoguanine (8-oxoG) and 2,6-diamino-4-hydroxy-5-N-methylformamidopyrimidine (Fapy) from DNA. The potential for OGG1 inhibitors for use as a monotherapy in cancer treatment has been shown by the increased sensitivity of cells from patients with MMR deficiency that accumulate high levels of 8-oxoG [23], identifying a synthetic lethal relationship between MMR and BER.

Several efforts for developing small molecule inhibitors to OGG1 have been made. A hydrazide compound (O8) inhibited OGG1 with an IC_50_ of 0.22–0.35 μM, and had lesser effect on NEIL1 and NTH1 glycosylases that have overlapping substrate specificity [24]. Another inhibitor, SU0268, had a lower IC_50_ (59 nM), good permeability and low cytotoxicity on normal cells where an increase in genomic 8-oxoG was demonstrated [25]. A dual inhibitor (SU0383) was subsequently developed that would also inhibit MutT human homolog-1 (MTH1; aka NUDT) whose major substrate is 8-oxoG nucleotides [26]. By inhibiting both enzymes that clear genomic 8-oxoG and oxidized bases from the nucleotide pool, the increased oxidation load would tip the cancer cells into apoptosis, in the same way as proposed by MTH1 inhibitor alone [27,28]. The effect of these OGG1 inhibitors in cellular and animal cancer models is currently being pursued.

A small molecule OGG1 inhibitor, TH5487, with a different mechanism has recently been developed that inhibits the binding of OGG1 to 8-oxoG rather than catalysis [29]. TH5487 is a promising anti-inflammatory drug as it prevents the transcription of inflammatory response genes through deficiencies in 8-oxoG repair [29]. However, the utilisation of TH5487 within cancer cell models has yet to be reported.

### NEIL1 inhibitors

Identification of other DNA glycosylases as potential drug targets was investigated using an siRNA screening approach which looked for increased sensitivity to uracil incorporation by various folate and nucleotide analogues. NEIL1 (and OGG1) siRNA-mediated depletion increased the cytotoxicity of thymidylate synthetase inhibitors in U2OS cells [30]. In the same study, depletion of NTH1, MPG, SMUG1 and TDG were only weakly synergistic. Furthermore, siRNA knockdown of NEIL1 (and also PARP1) was synthetic lethal with FANCG loss (involved in DNA interstrand cross-link repair), sporadic mutations of which occur in several cancers [31]. The development of inhibitors to NEIL1 has led to identification of purine analogues, specifically derivatives of 2-thioxanthine (2TX), that are irreversible inhibitors to NEIL1 and effective *in vitro* [32,33]. However, the development of NEIL1 inhibitors is still in its infancy and we await results using appropriate cancer cell and animal models.

### APE1 and AP site inhibition

APE1 is an essential enzyme in BER that recognises and incises AP sites creating SSBs, but it also has 3′-phosphodiesterase activity that can remove terminal lesions such as phosphoglycolate that are formed following certain chemotherapy treatments and ionising radiation [34,35]. Thus inhibiting APE1 function is an attractive strategy to increase efficacy of radiotherapy, chemotherapy drugs and overcoming drug resistance. MX (or TRC102) does not inhibit APE1 directly, but can bind and modify AP sites making them refractory to APE1 binding. MX can also block the lyase activity of bifunctional glycosylases. MX has completed phase I clinical trials for use as a chemosensitizing agent with the antifolate pemetrexed in solid tumours [36] and with fludarabine for lymphomas, the outcome being that the combinations were well tolerated [37]. Other phase I and phase II studies are ongoing.

Several APE1 inhibitors have been generated and explored, although problems with permeability or poor potentiation have been reported (reviewed by [38]). For example, the inhibitor CRT0044876 with an IC_50_ of ∼3 µM reduced survival of HT1080 fibrosarcoma cells in combination with methylmethanesulfonate (MMS) and TMZ, but not to ionizing radiation [39]. The APE1 inhibitor compound III (API3) with an IC_50_ of 2–12 µM also sensitized HeLa cells effectively to MMS and TMZ, and was tolerated in mice [40]. However, a more suitable inhibitor is still needed for use in cancer therapy. Interestingly, APE1 inhibition has been found to be synthetically lethal in PTEN deficient melanoma cancer cell lines [41] and also with DSB repair proteins, specifically in BRCA1/2 and ATM-deficient cell lines [42]. Additionally, the interaction with nucleophosmin (NPM1) appears to be important in the response to platinum-based drugs as high expression levels of APE1 and NPM1 predict poor response to treatment. Thus, targeting APE1 activity, or its interaction with NPM1, might sensitize certain cancers expressing higher levels of these proteins [43]. These strategies, however, require further investigation.

APE1 is also known as Redox factor 1 (Ref1), and can respond to altered oxidative states by its cysteine-rich redox domain where it activates the DNA binding of certain transcription factors (e.g. NF-κB, p53, STAT3 and HIF-1α) [44]. Inhibitors have been identified to target the redox function of APE1 (reviewed by [38]). For example, E3330 (an NF-κB inhibitor) and Gossypol/AT101 (BCL2 inhibitors) can bind APE1 making it less redox active [45,46]. These agents can induce cytotoxicity as single agents [47] or in combination [46,48] in lung, lymphoma, prostate, adrenocortical and glioblastoma cancers, and more than 20 clinical trials are currently investigating their suitability.

### PNKP inhibitors

Polynucleotide kinase phosphatase (PNKP) has dual 5′-kinase and 3′-phosphatase activities on SSBs and DSBs. Imidopiperidines have been identified as non-competitive inhibitors to the DNA–PNKP complex specifically inhibiting the phosphatase reaction [49,50]. One such inhibitor, A12B4C3, with an IC_50_ of ∼10 µM acts as a radiosensitizer in response to densely ionising carbon ion irradiation in prostate cancer cells [51], and Auger-emitting radioimmunotherapy in human myeloid leukaemia cells [52]. Synthetic lethal partnerships of PNKP with PTEN in colon cancer cell lines, and with the protein tyrosine phosphatase SHP-1 in T-cell lymphoma cell line have been identified, and where the combination of deficiencies in these proteins with A12B4C3 is effective in cell killing [53,54]. Interestingly, methods of delivering inhibitors of PNKP directly to the tumour through micelle encapsulation of the drug have been described, which are capable of radiosensitizing colon cancer cells [55]. This could potentially avoid any sensitisation of normal cells in proximity to the tumour being treated.

### FEN1 inhibitors

The small molecule inhibitor of FEN1, NSC-281680 with an IC_50_ of 1.2 µM, sensitized MMR-proficient and deficient colon cancer cells to TMZ [56]. Whereas the FEN1 inhibitor, SC13 with an IC_50_ of 4.2 µM, supressed growth of breast cancer cell lines and also sensitised cells to cisplatin, 5-FU and TMZ [57]. Several synthetic lethal partners for FEN1 have been identified, including MRE11 and CDC4-deficient colorectal cancers [58], and BRCA1/2-deficient cells [59], which both respond to small molecule inhibitors of FEN1. Recently, FEN1 expression was found to be a predictive marker for resistance to tamoxifen in ERα-positive breast cancers, and that a novel FEN1 inhibitor (FENi#2) reduced breast cancer cell proliferation *in vitro*, even in tamoxifen resistant cell lines [60]. Thus, FEN1 inhibitors appear to show great potential.

### DNA polymerase β inhibitors

Polβ is a key player in BER and is therefore an attractive target for chemosensitization of cancer cells. Many inhibitors to Polβ have been developed since the 1990s, although unfortunately most are non-specific (e.g. also targeting other DNA polymerases) or not potent or soluble enough to enter the cell or to be used clinically (see review [61]). However, a small molecule inhibitor of Polβ, NSC666715 (with an IC_50_ of ∼4 µM), designed by *in silico* molecular docking blocks the strand-displacement activity of Polβ in LP-BER leading to AP site accumulation and S-phase cell cycle arrest in colorectal cancers [62]. NSC666715 also appeared to potentiate the effects of TMZ in inducing cellular senescence in these cell lines. More recently, Pro-13, an irreversible inhibitor of Polβ (and Polλ) with an IC_50_ of 0.4 µM demonstrated little cytotoxicity in HeLa cells, but had a large synergistic effect in combination with MMS [63]. Natamycin, an antibiotic/anti-fungal agent, has been shown to inhibit the strand displacement activity of Polβ (at 2–5 nM), and at higher (µM) concentrations inhibited both Polβ and LigI, and consequently reduced proliferation of androgen-depleted prostate cancer cell lines [64].

An alternative strategy to directly inhibiting Polβ is through targeting protein stability. We have described that the deubiquitylating enzyme ubiquitin specific protease 47 (USP47), controls the cellular protein levels of Polβ through ubiquitylation-dependent degradation [65]. An siRNA knockdown of USP47 led to reduced Polβ protein levels and increased sensitivity of HeLa cells to MMS and hydrogen peroxide. Importantly, USP47 shares structural similarity to USP7, which plays a major role in stabilizing the p53 tumour suppressor protein and where inhibitors against USP7 are actively being sought. Indeed, inhibitors to both enzymes have been reported [66,67]. However, improvements in drug potency, solubility and stability are now required prior to detailed examination of USP7/USP47 inhibitors in impacting on BER and cancer cell survival.

### DNA ligase inhibitors

The *ligIII* gene encodes a mitochondrial form of LigIIIα which is essential for cell survival, whereas the nuclear form is in complex with XRCC1 and is dispensable as LigI can compensate for its cellular role [68–70]. As with the DNA polymerases, obtaining inhibitors that are specific and potent has been difficult (comprehensively reviewed by [71]). Structure-based design of inhibitors have produced a series of compounds that are specific to LigI (L82, L82-G17), LigI and LigIIIα (L67) or all DNA ligases (L189) with IC_50_ of ∼10 μM [72–74]. L82 was cytostatic, whereas L67 and L189 were cytotoxic in MCF7, HeLa and HCT116 cells. Interestingly, at subtoxic levels L67 and L189 were found to increase the sensitivity of MCF7 breast cancer cell lines to MMS or ionising radiation, but had no impact on sensitisation of normal breast cell lines. LigI is often elevated in cancer cells due to hyper proliferation, and also the levels of other DNA ligases may be dysregulated that may explain this apparent selectivity of the inhibitors [75]. Thus, ligase inhibitors have an important place in functional studies and cancer therapy, though not necessarily as a result of BER targeting.

### PARP inhibitors

**Figure 2:**
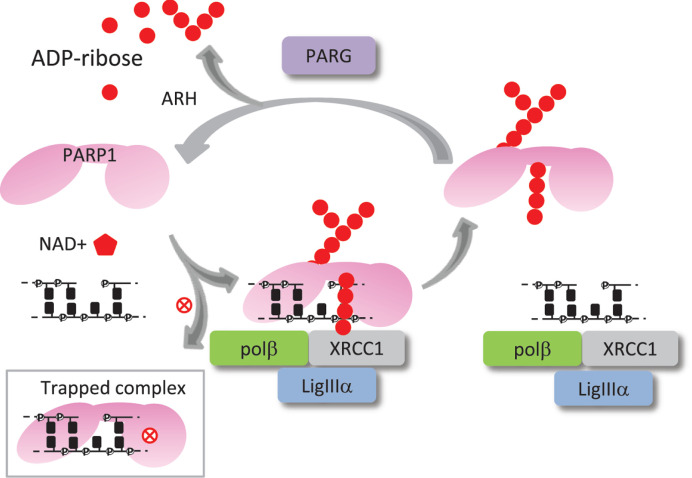
Functions of PARP1 and PARG during BER PARP1 (pink) recognises SSB/gap intermediates and uses NAD+ (red hexagon) to synthesise linear and branched chains of ADP-ribose units (red circles) to itself and/or other proteins. The BRCT1 domain of XRCC1 can bind poly(ADP-ribose), and make DNA contacts, allowing access for Polβ and LigIIIα to repair the break. Accumulation of PARP1 poly(ADP-ribosyl)ation causes PARP1 to be released through electrostatic repulsion. PARG subsequently cleaves the poly(ADP-ribose) chains allowing PARP1 to bind to additional SSB/gaps. The terminal ADP-ribose unit is refractory to PARG action, so ADP-ribose hydrolases (ARH) are needed for complete removal. PARPi (red cross) can lead to a trapped PARP–DNA complex that interferes with DNA replication.

PARP1 (and its associated backup enzyme PARP2) recognise gaps and SSBs in the DNA backbone and catalyse the addition of ADP-ribose units to itself or other proteins from cellular NAD+ (Figure 2). PARP inhibitors (PARPi) have been developed to inhibit PARP1, PARP2 (and PARP3) with nanomolar IC_50_ by binding to the NAD+ binding site in the catalytic domain, but can vary in selectivity among other PARP family members [76]. It should be noted that PARPi would also affect the response of PARP1 to DSBs and of PARP3 activity on recognition of both SSBs and DSBs [77–79]. The formation of poly(ADP-ribose) can relax chromatin structure and is recognised by a variety of poly(ADP-ribose) binding motifs located in a number of DNA repair factors and chromatin remodelling factors (reviewed by [80]). Importantly, XRCC1 is localised to DNA damage through its interaction with poly(ADP-ribose) and DNA mediated through sites on either side of its BRCT1 domain [81]. XRCC1 acts as a scaffold protein and provides a platform for Polβ, LigIIIα, PNKP, aprataxin and APLF binding. Thus, PARPi can disrupt the coordination of DNA repair proteins, chromatin accessibility and chromatin remodelling.

PARP1 was first demonstrated as a synthetic lethal partner to BRCA1 and BRCA2 mutations in breast cancer cells that are unable to effectively perform homologous recombination (HR) [82,83]. PARPi (olaparib, rucaparib, niraparib and talazoparib) have since been approved for use as monotherapy agents for BRCA-mutated breast, ovarian cancer, fallopian tube and peritoneal cancers. Many other synthetic lethal partners for PARPi have been identified and not just limited to HR, increasing the potential use of PARPi as a synthetic lethal agent [84]. Interestingly, there is evidence that PARPi can enhance the radiosensitivity of cell lines from different tumour types that appear to display proficient-HR mechanisms, including head and neck cancer, and glioblastoma [85–87]. Indeed, PARPi are currently in a large number of clinical trials for combination therapies as radio/chemosensitizers for a variety of other cancer types (e.g. prostate, gastric, haematological, lung, brain, head and neck, colorectal and advanced solid tumours). However, there is some debate as to whether sensitization occurs via BER or other PARP1-dependent functions [87–89].

The cytotoxicity of the inhibitors differs considerably (talazoparib >olaparib>veliparib) and depends on specificity, potency of catalytical inhibition, pharmacodynamic/kinetic properties and ‘trapping’ ability [90]. In the absence of auto(ADP-ribosyl)ation, PARP1 and PARP2 inhibited by talazoparib or olaparib remain bound to DNA in trapped complexes that cause PARP retention on chromatin (Figure 2) [91,92], whereas some inhibitors cause an allosteric change that releases the PARP from DNA (e.g. veliparib) [90]. An emerging problem with PARPi is the development of drug resistance largely through restoration of HR by secondary mutations in BRCA proteins or proteins that favour HR pathway choice (reviewed in [93]). PARP1 mutations and down-regulation also occur in PARPi-resistant cells [94]. Alterations in miRNA expression can produce similar outcomes on HR/PARP activity. Interestingly, poly(ADP-ribose) glycohydrolase (PARG) loss can also cause drug resistance presumably by increasing poly(ADP-ribosyl)ation levels [95]. Thus, improving cytotoxicity through drug design and overcoming drug resistance will improve the efficacy of PARPi use in the future.

### PARG inhibitors

PARG is an essential protein required for the breakdown of poly(ADP-ribose) chains, and the recycling of PARP1 (and PARP2) for co-ordinating additional BER activity (Figure 2). Like PARPi, PARG loss is synthetic lethal with BRCA2 mutations as well as with other partners involved in HR, including BRCA1, PALB2, FAM175A and BARD1 [96,97]. The specific PARG inhibitor (PARGi), PDD00017273, led to an increase in the number of stalled replication forks requiring HR for repair, and ultimately enhanced death of MCF7 cells. The PARGi was also similarly effective as PARPi in radiosensitising MCF7 cells, but the mechanism of sensitization differed as this occurred through altering mitosis [98].

PARG activity can also regulate the activity of a number of transcription factors [99]. For example, PARG activity can increase the expression of androgen receptor by removing inhibitory poly(ADP-ribosyl)ation from the transcriptional coactivator KDM4D. PARGi, PDD00017272, enhanced the effect of androgen ablation on prostate cancer cell lines, by further reducing androgen receptor signalling in addition to increased cytotoxic breaks arising from the inhibition of BER [100]. The development of other PARGi (e.g. COH34, JA2-4 and JA2131) to exacerbate replicative stress are proving promising as synthetic lethal agents, chemosensitizers and for re-sensitizing PARPi-resistant cells [101,102].

## Concluding remarks

BER is a critical cellular DNA repair pathway responding to DNA base damage and SSBs, and we describe here some studies where BER inhibitors have shown promise as radio/chemosensitizers in several cancers or form synthetic lethal partnerships with common cancer mutations. PARPi in particular have achieved the greatest success as approved monotherapy agents and are also in clinical trials as radio/chemosensitizers. The AP site inhibitor MX and APE1/BCL2 redox inhibitors Gossypol/AT101, are also currently in clinical trials but other inhibitors appear to have fallen short thus far. So, why has BER inhibitor development and clinical use been so challenging?

A large number of the BER proteins are embryonic lethal in knockout mice (e.g. APE1, Polβ, LigIII, LigI and FEN1) suggesting that inhibitors to these proteins might be toxic to normal cells. The DNA glycosylases, on the other hand, are not embryonic lethal in mice (with the exception of TDG) as there is a degree of redundancy among these enzymes, which can therefore diminish the impact of any targeted drug. In addition, backup repair pathways exist for several DNA lesions processed by BER, namely, HR, MMR, nucleotide incision repair and nucleotide excision repair [103–106], which can also potentially reduce inhibitor efficacy. The development of specific inhibitors to many of the BER enzymes has been arduous because they belong to families of functionally diverse but structurally similar enzymes. Nevertheless, new strategies for designing inhibitors based on structural data and in-depth molecular mechanisms have made great advances in recent years, and which should be continually explored.

An impressive advancement of immunotherapy as an approach for effective cancer treatment has been made in recent years. Interestingly, recent reports suggest that PD-L1 expression is negatively correlated with BER gene expression, including OGG1 and APE1 [107], and that anti-PD-1 therapy in combination with ionising radiation is stimulated by PARPi in colorectal cancer models [108]. These intriguing findings have opened up new exciting therapeutic opportunities, and which nevertheless support that there should be ongoing research into targeting the BER both as a monotherapy but also as a combinatorial therapy for cancer treatment.

## Summary

- SP-BER and LP-BER are vital in excising damage to bases and repairing SSB in DNA, thereby reducing mutagenesis.
- DNA damage caused by radiotherapy and many chemotherapeutics is required to exceed BER capacity for effectiveness.
- Targeting BER enzymes can increase radio/chemosensitivity, re-sensitize drug resistant cancers or form part of a synthetic lethal combination with cancer mutations.
- PARP inhibitors have proved successful in clinical trials, inhibitors that affect APE1 functions have progressed to phase II/III clinical trials, and several other BER enzymes remain promising targets.

## Open Access

Open access for this article was enabled by the participation of University of Liverpool in an all-inclusive *Read & Publish* pilot with Portland Press and the Biochemical Society under a transformative agreement with JISC.

## Abbreviations

8-oxoG: 7,8-Dihydro-8-oxoguanine
APE1: AP endonuclease 1
AP: apurinic/apyrimidinic
BER: base excision repair
dRP: 5′-deoxyribosephosphate
Fapy: 2,6-diamino-4-hydroxy-5-N-methylformamidopyrimidine
FEN1: Flap endonuclease 1
HR: homologous recombination
LigI: DNA ligase I
LigIIIα: DNA ligase IIIα
MMR: mismatch repair
MMS: methylmethanesulfonate
MX: methoxyamine
NEIL: endonuclease VIII-like
NPM1: nucleophosmin
OGG1: 8-oxoguanine DNA glycosylase
PARG: poly(ADP-ribose) glycohydrolase
PARP1: poly(ADP-ribose) polymerase 1
PARPi: PARP inhibitor
PNKP: polynucleotide kinase phosphatase
Polβ: DNA polymerase β
Ref1: Redox factor 1
SSB: single strand break
TMZ: Temozolomide
UNG: uracil DNA glycosylase
XRCC1: X-ray repair cross-complementing protein 1
