# Correction: Base excision repair and its implications to cancer therapy

**DOI:** 10.1042/EBC20200013_COR

**Published:** 2020-07-29

Grundy, G.J. and Parsons, J.L. (2020) Base excision repair and its implications to cancer therapy. *Essays Biochem.* https://doi.org/10.1042/EBC20200013

During the production process errors were introduced into Table 1. The correct version of Table 1 appears here.

**Table 1:** DNA glycosylases: substrates, inhibitors and synthetic lethal partners

DNA Glycosylase	Substrate	Inhibitor	Synthetic lethal partner
*Monofunctional*			
**UNG**			
Uracil DNA glycosylase	Uracil		APOBEC3B
**SMUG1**			
Single-strand selective monofunctional uracil DNA glycosylase	Uracil, 5-formyluracil, 5-hydroxyuracil and 5-hydroxymethyluracil		
**TDG**			
G/T Mismatch specific thymine DNA glycosylase	5-Formylcytosine and 5-carboxylcytosine in CpG. G:T and U:T mismatches		
**MBD4**			
methyl-CpG binding domain protein 4	G:T mismatches within methylated and unmethylated CpG sites. Uracil or 5-fluorouracil in G:U mismatches		
**MPG**			
N-methylpurine DNA glycosylase	3-methyladenine, and 7-methylguanine		
**MUTYH**			
Adenine DNA glycosylase	7,8-Dihydro-8-oxoguanine (8-oxoG):adenine		
*Bifunctional*			
**NTH1**			
Endonuclease III-like protein 1	Oxidised pyrimidines, thymine glycol		
**OGG1**			
8-oxoguanine DNA glycosylase 1	8-OxoG, formamidopyrimidine (Fapy)G	O8	MMR deficiency
		SU0268	
		TH5487	
*Endonuclease VIII-like*			
**NEIL1**	Thymine glycol, Fapy, 5-hydroxyuracil	2TX	FANCG
**NEIL2**	5-Hydroxyuracil		
**NEIL3**	Spiroiminodihydantoin and guanidinohydantoin		

